# Sex differences in hepatic one-carbon metabolism

**DOI:** 10.1186/s12918-018-0621-7

**Published:** 2018-10-24

**Authors:** Farrah Sadre-Marandi, Thabat Dahdoul, Michael C. Reed, H. Frederik Nijhout

**Affiliations:** 1 Mathematical Biosciences Institute, The Ohio State University, Columbus, 43210 OH USA; 2 Department of Mathematics, Cal-State Fullerton, Fullerton, 92831 CA USA; 3 Department of Mathematics, Duke University, 120 Science Drive, Box 90320, Durham, 27708 NC USA; 4 Department of Biology, Duke University, Durham NC 27705, USA

**Keywords:** One-carbon metabolism, Mathematical model, Sex differences, Regulation

## Abstract

**Background:**

There are large differences between men and women of child-bearing age in the expression level of 5 key enzymes in one-carbon metabolism almost certainly caused by the sex hormones. These male-female differences in one-carbon metabolism are greatly accentuated during pregnancy. Thus, understanding the origin and consequences of sex differences in one-carbon metabolism is important for precision medicine.

**Results:**

We have created a mathematical model of hepatic one-carbon metabolism based on the underlying physiology and biochemistry. We use the model to investigate the consequences of sex differences in gene expression. We give a mechanistic understanding of observed concentration differences in one-carbon metabolism and explain why women have lower S-andenosylmethionine, lower homocysteine, and higher choline and betaine. We give a new explanation of the well known phenomenon that folate supplementation lowers homocysteine and we show how to use the model to investigate the effects of vitamin deficiencies, gene polymorphisms, and nutrient input changes.

**Conclusions:**

Our model of hepatic one-carbon metabolism is a useful platform for investigating the mechanistic reasons that underlie known associations between metabolites. In particular, we explain how gene expression differences lead to metabolic differences between males and females.

**Electronic supplementary material:**

The online version of this article (doi:10.1186/s12918-018-0621-7) contains supplementary material, which is available to authorized users.

## Background

There are significant sex differences in one-carbon metabolism (OCM) and these differences are accentuated in pregnancy [1–3]. Women in the child-bearing years exhibit lower plasma homocysteine (Hcy) [4], higher betaine and choline [5], and lower S-andenosylmethionine (SAM) [6]. Various enzymes in OCM are upregulated or downregulated in women relative to men [4, 7, 8]. For example, phosphatidylethanolamine N-methyltransferase (PEMT) is upregulated by estrogen [4, 9]. Furthermore, insulin and glucose affect some enzymes of OCM [10] and change during pregnancy [1]. All of these results suggest that a mechanistic understanding of how enzymatic differences in women affect OCM is important for precision medicine.

**Fig. 1:**
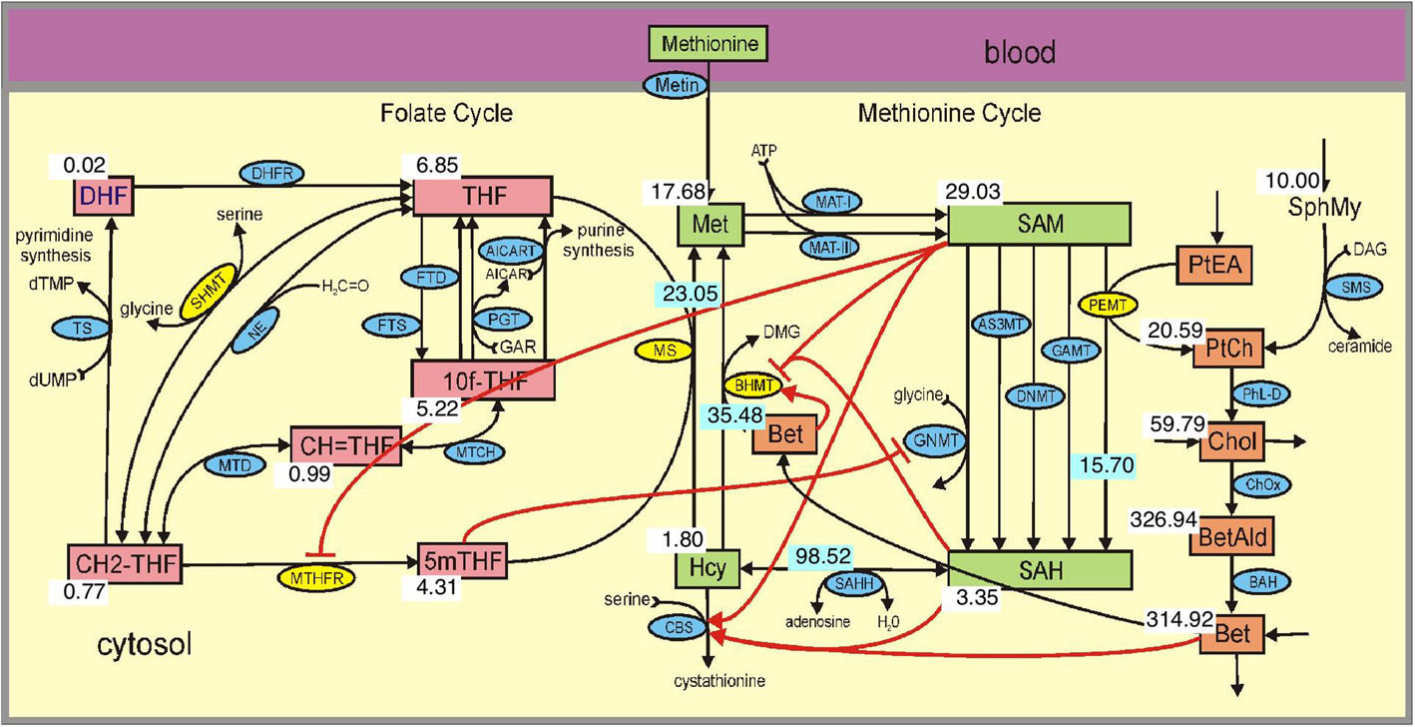
One-Carbon metabolism for the male. Substrates are indicated by rectangular boxes, green in the methionine cycle and red in the folate cycle. Each black arrow represents a biochemical reaction and the blue and yellow ellipses on the arrows contain the acronyms of the enzymes that catalyze the reactions. The yellow ellipses indicate the enzymes that are up- or down-regulated in females (see Table 1). Each red arrow is a long-range allosteric influence, either activation (arrow) or inhibition (bar). The numbers next to the substate boxes indicate the normal steady state values of the concentrations in micromolar. The numbers in the blue boxes are flux values in micromolar/hour. Substrate abbreviations: Met, methionine; SAM, S-adenosylmethionine; SAH, S-adenosylhomocysteine; Hcy, homocysteine; 5mTHF, 5-methyltetrahydrofolate; THF, tetrahydrofolate; 10fTHF, 10-formyltetrahydrofolate; DHF, dihydrofolate; CH2-THF, 5,10-methylenetrahydrofolate; CH=THF, 5,10-methenyltetrahydrofolate; SphMY, sphimgomyelin; PtEA, phosphotidylethanolamine; Cho, choline; Bet-Ald, betaine aldehyde; Bet, betaine. Enzyme abbreviations: AICAR(T), aminoimidazolecarboxamide ribonucleotide (transferase); FTD, 10-formyltetrahydrofolate dehydrogenase; FTS, 10-formyltetrahydrofolate synthase; MTCH, 5,10-methylenetetrahydrofolate cyclohydrolase; MTD, 5,10-methylenetetrahydrofolate dehydrogenase; MTHFR, 5,10-methylenetetrahydrofolate reductase; TS, thymidylate synthase; SHMT, serine hydroxymethyltransferase; PGT, phosphoribosyl glycinamidetransformalase; DHFR, dihydrofolate reductase; NE, nonenzymatic interconversion of THF and 5,10-CH2-THF; MAT-I,methionine adenosyl transferase I; MAT-III, methionine adenosyl transferase III; GNMT, glycine N-methyltransferase; AS3MT, arsenic methyltransferase; PEMT, phosphotidylethanolamine methyltransferase; GAMT, gunadino-acetate methyltransferase; DNMT, DNA-methyltransferase; SAHH, S-adenosylhomocysteine hydrolase; CBS, cystathionine *β*-synthase; MS, methionine synthase; SMS, sphingomyelin synthase; PhL-D, phospholipase D; ChOx, choline oxidase; BAH, betaine aldehyde dehydrogenase; BHMT, betaine-homocysteine methyltransferase

The reaction diagram for the folate and methionine cycles in OCM is very complicated consisting of loops within loops (see Fig. 1 below). Furthermore, many substrates in the network influence, through allosteric binding, the activity level of enzymes at distant locations in the network. We have shown that these long-range regulatory mechanisms are extremely important for stabilizing important concentrations and reactions (like DNA methylation and cell replication) against large changes in amino acid inputs due to meals and the environmental availability of B-vitamins [11–16]. Many (but not all) of these long-range regulations are indicated by the red arrows in Fig. 1. A consequence of these allosteric interactions is that it is almost impossible to guess the effects of the up- or down-regulation of a particular enzyme. These are systems properties of the whole network and to understand them one needs a mathematical model of OCM, based on the real underlying biochemistry and biology, and machine computation.

**Table 1:** Liver betaine concentration and BHMT/CBS activity data taken from [8]

25CG Diet	Liver Betaine *μ*mol/g	Liver BHMT Activity	Liver CBS Activity
+0% Bet	0.6	1.1	4.9
+0.05% Bet	1.7	1.5	5.8
+0.1% Bet	2.4	1.8	5.2
+0.2% Bet	2.8	2.7	5.3

Male rats were fed 25% casein diet (25C) diets with betaine for 10 days to investigate the dose-dependent effects of supplementation on hyperhomocysteinemia induced by guanidinoacetic acid (GAA) addition and choline deprivation

**Fig. 2:**
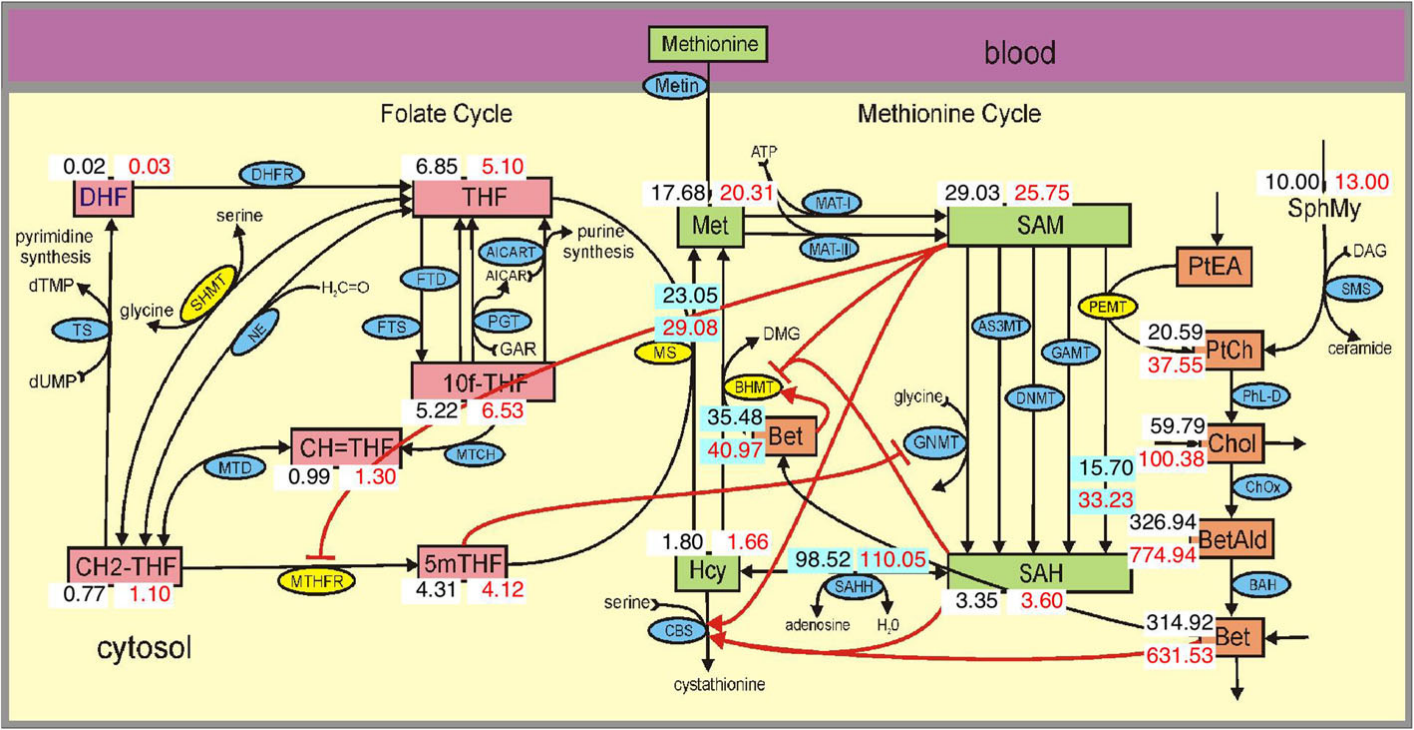
One-Carbon metabolism for the female. Substrates, enzymes, and allosteric interactions are as in Fig. 1. The six female adaptations from Table 3 were put in the model. The red numbers indicate the steady state concentrations of metabolites for the female in micromolar. The black numbers are the male concentrations from Fig. 1 for comparison. Females have lower SAM, higher SAH, lower Hcy, and much higher Chol and Bet. Females have higher remethylation flux and much higher PEMT flux, as well as higher flux overall around the methionine cycle

In [17] we created a mathematical model of folate and methionine metabolism to study the regulation of competing methyl transferases. In this study we extend the mathematical model by adding the pathway for the synthesis of choline and betaine (the orange boxes on the right in Fig. 1) and two allosteric regulations by betaine. The details of these changes are given in the Methods and Fig. 1 shows the metabolite concentrations for a male in the model. In the first section of Results, we describe the enzymatic changes in females and show in Fig. 2 the resulting concentrations of metabolites. In the following sections we discuss choline and betaine, SAM, folate, homocysteine (Hcy), and the effects of folate deficiency, methylenetetrahydrofolate reductase (MTHFR) polymorphisms, and variations in methionine (Met) input. We compare our results to clinical and experimental studies and discuss the causal mechanisms by which the gene expression or enzyme activity changes in women lead to the metabolite changes.

## Methods

The schematic diagram of the mathematical model is shown in Fig. 1. The pink boxes in the folate cycle and the green boxes in the methionine cycle indicate metabolites whose concentrations can change in the mathematical model (variable metabolites). The model described here is an expansion of a model previously described in [17]. An additional pathway for choline and betaine synthesis has been added, where the variable metabolites are indicated by the orange boxes. The arrows represent biochemical reactions and the blue and yellow ellipses show the acronyms of the enzymes catalyzing the reactions. The yellow ellipses indicate the enzymes that are substantially up- or down-regulated in females. Full names of the enzymes and substrates are in the legend of Fig. 1. The allosteric interactions crucial for our investigations in this study are indicated by red arrows. There are other allosteric interactions in the model that are not included in Fig. 1. For example, S-andenosyl-homocysteine (SAH) inhibits each of the methyltransferases and SAM affects both of the enzymes that synthesize it from Met.

We follow standard nomenclature and refer to the sum of the fluxes of the 5 methyltransferase reactions from SAM to SAH as the transmethylation flux. The sum of the methionine synthase (MS) and betaine-homocysteine methyltransferase (BHMT) reaction fluxes is called the remethylation flux, and the cystathionine *β*-synthase (CBS) reaction flux is called the transsulfuration flux. Note that, at steady state, the remethylation flux plus the methionine input flux must equal the transmethylation flux and the methionine input flux must equal the transsulfuration flux. All concentrations and *K*_*m*_ values are in micromolar. All fluxes are in micromolar/hour.

The complete mathematical model consists of 17 differential equations for 17 metabolites. Each differential equation tracks the concentration change of the metabolite, by adding the sum of the rates of the arrows coming into that variable and by subtracting the sum of the rates of the arrows leaving the variable. Contributions of the mitochondria to one-carbon metabolism have been studied in [18], the transulfuration pathway was studied in [19] and whole body folate and methionine metabolism was studied in [20]. The model described here (in this “Methods” section) is a liver model for males and will be referred to as “the male model;” adaptations in females are discussed in “Sex differences” section.

We begin by describing briefly our modeling approach and technique. Our goal is to understand biological phenomena and we use mathematical models as our tools. Our models are based on the underlying physiology and biochemistry, but that doesn’t mean that they contain all the details of the enzymes kinetics that are known. Quite to the contrary, we believe in constructing relatively sparse models so that we can experiment with them to see if they are sufficient to explain the experimental and clinical data. If not, then we add more detailed mechanisms as necessary. So, for example, we have not included the folate cycle in the mitochondria or the transsulfuration pathway. For the individual reactions, we start with Michaelis-Menten kinetics and then add the allosteric interactions that we believe are important for the phenomena that we wish to understand. We choose *K*_*m*_ values from the ranges in the literature. Then we adjust the *V*_*max*_ values so that the metabolite concentrations at steady state are consistent with the concentrations in the literature. In fact, the *V*_*max*_ values are proportional to enzyme expression levels and enzyme expression levels vary by up to 25% from individual to individual [21, 22]. Thus, we view our model as a model for an “average male,” and we check to make sure that the qualitative behavior of the model remains the same even if parameters vary widely. Our goal is to explain how the qualitative behavior arises from the systems biology of the network, for example, “Why do women have lower Hcy than men?”

The rest of this section presents the new substrates and reactions that have been added to the model in [17] in order to study sex differences in OCM. Full details of the complete model are available in the Additional file 1.

### Phophotidylethanolamine methyltransferase (PEMT)

PEMT is an enzyme of the endoplasmic reticulum and it has very complicated kinetics [23] that are probably highly dependent upon the membranous structure and lipid content (e.g. PE) in the vicinity of the enzyme. For our purposes here it is sufficient to assume a simple Michaelis-Menten form for the dependence on PE:

$$\begin{aligned} V_{\text{PEMT}}&([SAM],[SAH],[PE]) \\&\,=\, \!\left(\frac{V_{max}[SAM]}{(K_{m} + [SAM])\left(1 + \frac{[SAH]}{K_{i}}\right)}\right)\left(\frac{25*[PE]}{\left(K_{m} + [PE]\right)}\right). \end{aligned} $$

The inhibition by SAH is non-competitive [24]. We choose *K*_*m*_=18.2*μ*M for SAM and *K*_*i*_=3.8*μ*M for SAH as indicated in [25], *K*_*m*_=5000*μ*M for PE as indicated in [23], and *V*_*max*_=98*μ*M/hr. As explained above, we adjust the *V*_*max*_ so that the downstream metabolites have concentrations that are consistent with the literature. If we chose somewhat different *K*_*m*_ values, we would choose a somewhat different *V*_*max*_ value so that the flux would be similar. Thus our qualitative results are not sensitive to modest changes in *K*_*m*_ values.

### Sphyingomyelin synthase (SMS)

The velocity of the SMS reaction is given by

$$\begin{array}{@{}rcl@{}} V_{\text{SMS}}\left([SphMy]\right) & = & \frac{V_{max}[SphMy]}{\left(K_{m} + [SphMy]\right)}. \end{array} $$

where SphMy abbreviates sphingomyelin. We choose *K*_*m*_=20*μ*M as indicated in [26, 27] and *V*_*max*_=30*μ*M/hr.

### Phospholipase-D (PhL-D)

The velocity of the PhL-D reaction is given by

$$\begin{array}{@{}rcl@{}} V_{\text{PhL-D}}\left([PC]\right) & = & \frac{V_{max}[PC]}{\left(K_{m} + [PC]\right)}. \end{array} $$

where PC abbreviates phosphotidylcholine. We choose *K*_*m*_=400*μ*M for PC as indicated in [28] and *V*_*max*_=525*μ*M/hr.

### Choline oxidase (ChOx)

The velocity of the ChOx reaction is given by

$$\begin{array}{@{}rcl@{}} V_{\text{ChOx}}([Chol]) & = & \frac{V_{max}[Chol]}{(K_{m} + [Chol])} \end{array} $$

where Chol abbreviates choline. We choose *K*_*m*_=200*μ*M for choline, which is in the middle of the range found in [29], and *V*_*max*_=125*μ*M/hr.

### Betaine aldehyde dehydrogenase (BAH)

The velocity of the BAH reaction is given by

$$\begin{array}{@{}rcl@{}} V_{\text{BAH}}([BetAld]) & = & \frac{V_{max}[BetAld]}{(K_{m} + [BetAld])} \end{array} $$

where BetAld abbreviates betaine aldehyde. We choose *K*_*m*_=250*μ*M for BetAld, which is in the middle of the range found in [30], and *V*_*max*_=45*μ*M/hr.

### Betaine-homocysteine methyltransferase (BHMT)

The velocity of the BHMT reaction has three factors. The first factor is simply Michaelis-Menten kinetics for Hcy and betaine (Bet), with $K_{m}^{hcy} = 12\mu $M [31] and $K_{m}^{bet} = 2000\mu $ [32]. The second factor is the inhibition of BHMT by SAM and SAH, derived by non-linear regression on the data of [33], and scaled so that it equals 1 at the normal male steady state. The third factor is the activation of BHMT by betaine. BHMT mRNA has been shown to have up to a 3 fold increase with betaine supplementation [7]. In [8] it was shown that betaine supplementation causes a 45% increase in liver BHMT activity, Table 1 column 3. This was put into the model by assuming a linear increase in activity as the Bet concentration rises with a slope of 16%. The effect is scaled so that it equals 1 at the normal male steady state of 315 *μ*M for betaine.

$${\begin{aligned}  V_{\text{BHMT}}&\left([Hcy],[Bet],[SAM],[SAH]\right)\\ = & \left (\frac{V_{max}[Hcy][Bet]}{\left(K_{m}^{hcy} + [Hcy]\right)\left(K_{m}^{bet} + [Bet]\right)}\! \right) \cdot \left (\frac{e^{-.0021\left([SAM] + [SAH]\right)}}{ e^{-.0021(32.3)} }\right)\\ & \cdot \left(1 + \frac{(0.16)*\left([Bet]-315\right)}{315} \right). \end{aligned}} $$

### Cystathionine *β*-synthase (CBS)

The kinetics of CBS are standard Michaelis-Menten with *K*_*m*_=1000*μ*M for Hcy taken from [34]. The second term is the activation of CBS by SAM and SAH. The form of the activation was derived by non-linear regression on the data in [35, 36] and scaled (by the third term) so that it equals 1 when the system is at the normal steady state. The fourth term is the activation of CBS by betaine. Table 1, column 4, showing data from [8], suggests that a betaine increase can activate liver CBS by up to 18%.

$$\begin{aligned} V_{\text{CBS}}&([Hcy],[SAM],[SAH],[Bet]) \\ = & \left (\frac{V_{max}[Hcy]}{K_{m} + [Hcy]} \right) \left (\frac{(1.2)([SAM] + [SAH])^{2}}{(30)^{2} + ([SAM + SAH])^{2}} \right)\\ & \cdot \left (\frac{(1.2)(32.3)^{2}}{(30)^{2} + (32.3)^{2} }\right)^{-1}\\ & \cdot \left(1 + \mathcal{H}([Bet]-315)\frac{(0.2)*([Bet]-315)}{10 + ([Bet]-315)} \right). \end{aligned} $$

$\mathcal {H}([Bet]-315)$ is the Heaviside function which is equal to zero when the Bet concentration is at or below steady state (315 *μ*M) and equals one otherwise. Thus, the fourth term equals one when betaine is below 315 and increases up to 1.2 as betaine concentration rises.

We note that once one has the concentrations of the metabolites, then one obtains the metabolite fluxes by putting the concentrations into the above formulas.

The C677T and A1289C polymorphisms of MTHFR reduce the activity of MTHFR by 70% and 32%, respectively [37, 38]. In the “Vitamin deficiencies and polymorphisms.” section we test the effects of these polymorphisms by multiplying the *V*_*max*_ value of MTHFR by 0.3 and 0.68, respectively. Similarly, total folate concentration is a parameter in the model. We test the effects of different values of total folate by multiplying this parameter by appropriate scale factors.

## Results

### Sex differences

Five enzymes in one-carbon metabolism, BHMT, MS, MTHFR, serine hydroxymethyltransferase (SHMT), and PEMT, are differentially expressed in males and females and the differences are not small. In Fig. 1 these five enzymes have their acronyms in bright yellow ellipses, rather than blue ellipses. BHMT and MTHFR are downregulated in females and MS, SHMT, and PEMT are upregulated. Not surprisingly, these enzyme expression differences lead to concentration differences in choline, betaine, sphingomyelin, SAM, and Hcy, measured in experiments or in the clinic (see Table 2).

**Table 2:** Ratio of female values to male values for various enzyme expressions and concentrations in one-carbon metabolism

	Female/male	Animal	Reference
PEMT	2-2.3	Mouse, human hepatocytes	[68]
BHMT	0.54-0.6	Mice	[69]
MTHFR	0.37-0.93	Mice	[4]
MS	1.32-1.38	Mice	[4]
SHMT	1.89-2.44	Mice	[4]
Choline	1.44-2.75	Rats	[5]
Betaine	1.29-2.89	Rats and mice	[4, 5]
SphMy	1.18-1.3	Mice	[4]
SAM	0.82-1	Human, whole blood	[6]
Hcy	0.6-0.89	Humans	*, [4, 70–73]

The enzymes PEMT, BHMT, MTHFR, MS, and SHMT are up- or down-regulated in females. The concentrations of choline, betaine, sphingomyelin, SAM, and Hcy are higher or lower in females as indicated. The ranges for the ratios come from the cited literature. * indicates data from the NHANES 2005-2006 survey: wwwn.cdc.gov/nchs/nhanes/

The model described in Methods is a model for male one-carbon metabolism. The resulting concentrations and (some) velocities at steady state are shown in Fig. 1. To make a model for female one carbon metabolism, we multiply the *V*_*max*_ values of the enzymes PEMT, BHMT, SHMT, MS, MTHFR by the factors shown in Table 3. In each case, we chose the factor to be in the range found in experiments (see Table 2). In addition, we multiply the concentration of sphingomyelin by 1.3, the highest factor for SphMy shown in Table 2. We refer to these changes as “female adaptations.”

**Table 3:** Model parameter changes corresponding to female adaptations

PEMT	BHMT	SHMT	MS	MTHFR	SphMy
2.3	0.6	2.2	1.35	0.8	1.3

The *V*_*max*_ values of the five enzymes in yellow ellipses are multiplied by the factors shown and the concentration of SphMy is multiplied by a factor of 1.3

After making these changes, we ran the model to its new steady state. The resulting concentrations and velocities for the female model steady state are shown by the red numbers in Fig. 2. For convenient comparison, the male numbers from Fig. 1 are repeated in black. As one can see, choline and betaine are much higher in the female and homocysteine is lower in the female.

There are several interesting and important questions. The first is what are the mechanisms that cause these differences in enzyme expression levels in females? A good deal is known. The sex hormones estrogen, testosterone, and progesterone are found in both the males and females but in different proportions. Men have around 19 times more testosterone than women and women of childbearing age have up to 9 times more estrogen than men. Estrogen concentration increases even more throughout pregnancy [39]. It has been shown that estrogen impacts PEMT [40, 68]. The expression level of PEMT is increased by a factor of 2-2.3 in women of childbearing age compared to men [68]. In the methionine cycle, males express more BHMT (betaine-dependent Hcy remethylation), whereas females expressed more MS (folate-dependent Hcy remethylation) [4]. It has been shown that BHMT activity and MTHFR activity increase in response to testosterone and decrease after injection of estradiol in rats [41]. The change from testosterone to estrogen in females decreases the expression of BHMT by 40% [69]. Finally, progesterone upregulates sphingomyelin synthase [42], SMS, which is almost certainly the reason that females have higher sphingomyelin. Although MS is increased by 35% and SHMT by 111% in females [4], the reasons have not been determined, but these changes may also depend on the sex hormones.

Other hormones also cause sex dimorphism in liver enzymes. Waxman showed that the pulsatile release of growth hormone (GH) by the pituitary in males and the almost constant release in females cause differences in cytochrome P450 expression in rats [43]. DNA microarray analysis has not only confirmed this finding but has also shown that 27 of 37 female predominant genes in the liver were induced by continuous growth hormone treatment of male rats [44]. The authors conclude that GH regulated gene expression is a significant determinant of sexual dimorphic gene expression in the rat liver [44]. Variation in GH also plays an important role in a recent large scale model of male-female liver dimorphism by the Rozman group (described in the Discussion) [45–47].

The second question is what are the mechanisms by which the enzymes expression changes lead to the concentration changes. These questions are easy to state but not easy to answer, because the reaction network is complicated and the long range interactions, through which a substrate concentration at one location can affect enzymes at distant locations in the network (the red arrows in Figs. 1 and 2), make it difficult to guess the global effects of local enzyme changes. That is, these are systems biology questions that must be answered by appropriate in silico experiments on the mathematical model for the network. This is what we do in the succeeding sections and, as we shall see, the “reasons” for the metabolite concentration differences seen in the literature are sometimes surprising.

The third question is what are the physiological or evolutionary reasons that the enzyme changes and their consequences are beneficial to females? In the case of the upregulation of PEMT by estrogen, the answer is understood and we explain it in the “Discussion” section.

### Choline and Betaine

**Fig. 3:**
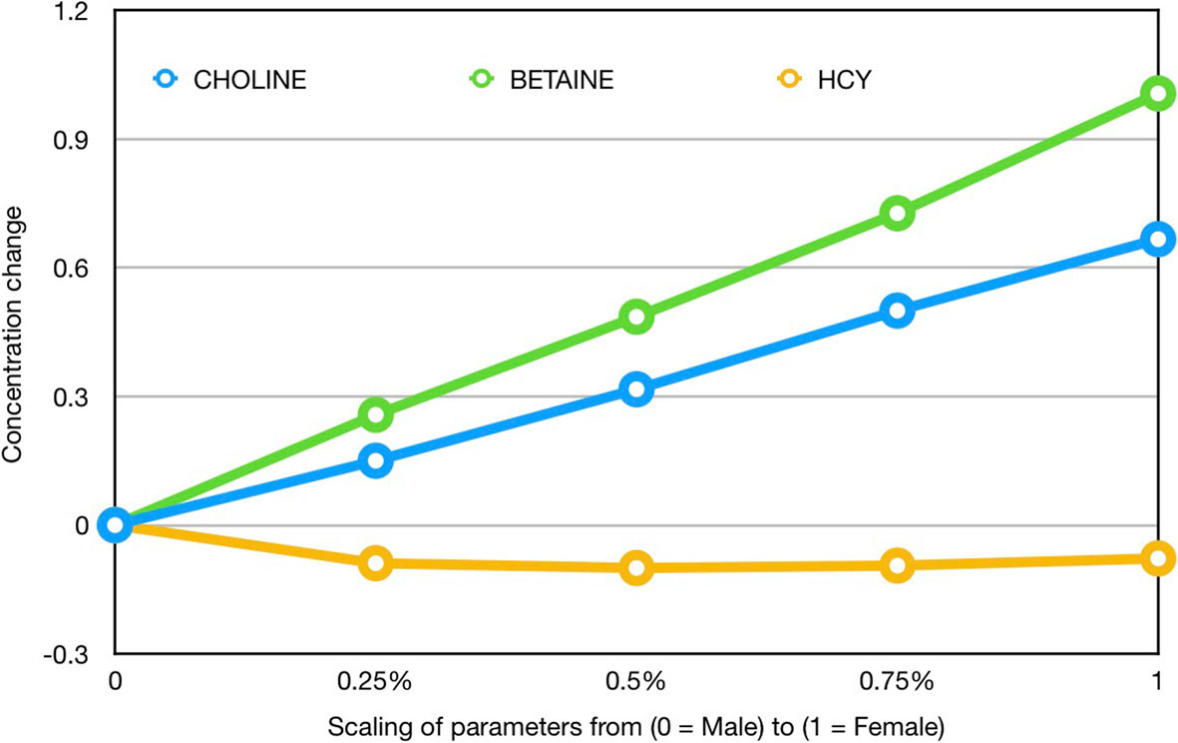
Choline and betaine simulation concentrations as all parameters transition from male to female. The six female adaptations in Table 3 are scaled from 0 (male) to 1 (female) and the choline, betaine, and homocysteine concentrations are indicated

Both choline and betaine, which are obtained from diet or by de novo synthesis in tissues, are important for maintaining health and choline deprivation can cause fatty liver, muscle damage, and organ dysfunction [48, 68]. And, as we see from Table 2 and Fig. 2, choline and betaine are much higher in females. It’s natural to assume that choline and betaine are higher because of the upregulation of PEMT by estrogen and, as we will see, that is partly true. First, we scale the five enzyme changes between the male (indicated by zero in Fig. 3) and the female (indicated by 1 in Fig. 3) and scale the change in SphMy. That way one can see explicitly how the concentrations of choline and betaine change as we scale from the male values to the female adaptations. As we transition from male parameters to female the concentrations of choline and betaine rise and homocysteine falls.

In order to investigate which sex differences cause the changes in choline and betaine we change them one at a time. Table 4 indicates how much choline and betaine rise, if we make one enzyme or SphMy have the female adaptation and the other five have the male values. The designation “ + all” indicates all the female adaptations from Table 3.

**Table 4:** Choline and betaine concentration values for different parameter changes

	Male	+PEMT	+BHMT	+SHMT	+MS	+MTHFR	+SphMy	+all
Choline	60	96	61	62	59	59	63	100
Betaine	315	381	546	379	299	290	333	632

One of the 6 parameters (as indicated by +) from Table 3 is changed to the female value while the other five are kept at the male values. “+ all” indicates all the female adaptations from Table 3

As indicated by Table 4, the sex differences in SHMT, MS, MTHFR, and SphMy have relatively small effects on choline and betaine. The primary reason for higher choline in females is the upregulation of PEMT and the primary reason for higher betaine in females is the downregulation of BHMT. Both of these changes are easy to understand by looking at Fig. 2.

### Why does folate raise SAM?

**Fig. 4:**
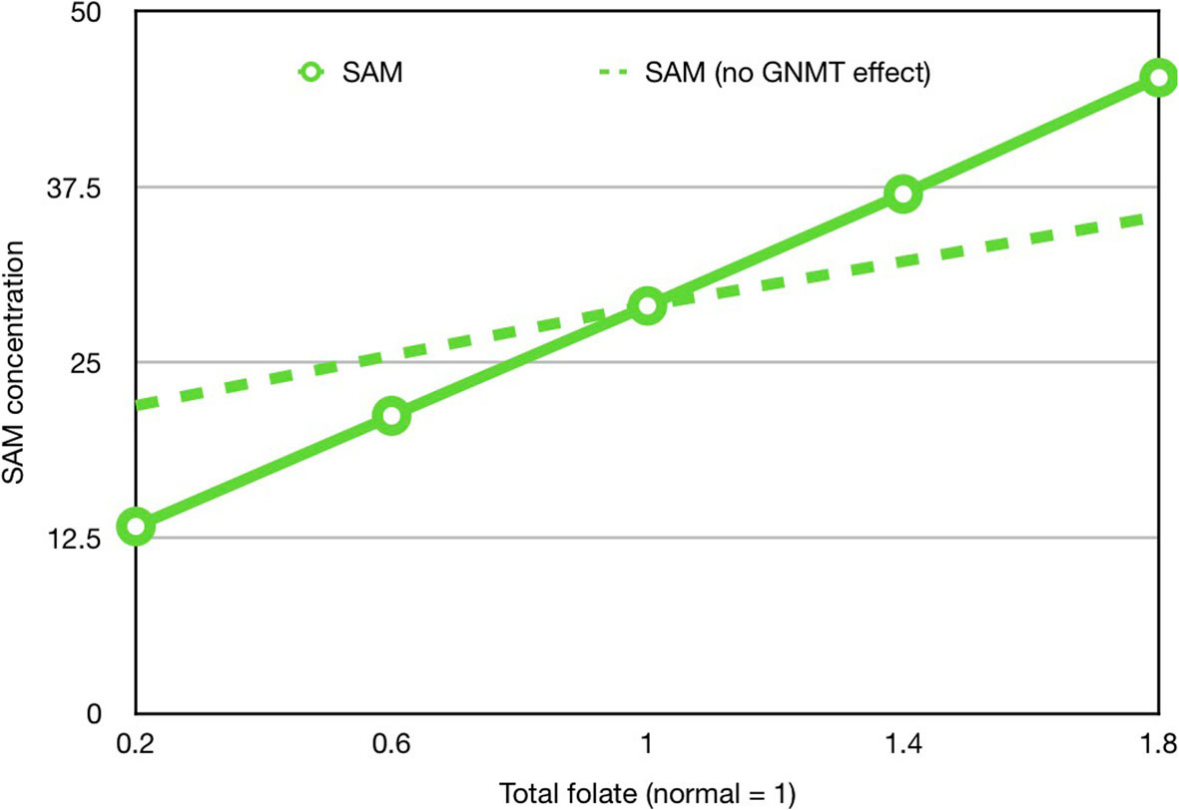
Why does folate make SAM go up? Total folate is indicated on the x-axis with normal folate = 1. The green curve shows that the SAM concentration increases linearly with total folate. The dashed curve shows the SAM concentration as a function of total folate if the inhibition of GNMT by 5mTHF is removed from the model. Most of the increase in SAM depends on the allosteric inhibition of GNMT by 5mTHF

In our earliest papers on modeling one-carbon metabolism we remarked that the SAM concentration increases as total liver folate increases and that the increase is linear over a wide folate range [18, 49]. This increase can be seen in the green curve in Fig. 4. But what is the mechanism? Notice that 5mTHF inhibits GNMT (see Fig. 1), so that if 5mTHF increases SAM should increase. If we remove the inhibition of GNMT by 5mTHF, then we obtain the dashed green curve in Fig. 4, showing that most, but not all, of the increase in SAM with higher folate is due to the inhibition of GNMT. The remaining increase is due to the fact that higher levels of 5mTHF drive the MS reaction faster at the new steady state, and thus raise the SAM concentration modestly (simulations not shown).

The inhibition of GNMT by 5mTHR was discovered by Wagner, Briggs, and Cook [50] and plays an extremely important role in making the methyltransferase reactions fairly independent despite the fact that they all use the same substrate, SAM [17]. The inhibition of GNMT is fairly complicated in that there are two binding sites for 5mTHF on GNMT. If one site is occupied by 5mTHF, then GNMT retains 50% activity and if both sites are bound GNMT loses all activity. These details, which are discussed thoroughly in [17], are in the model, but not indicated in Fig. 1.

Finally, we note that SAM is lower in females in the model (see Fig. 2). This corresponds well with observations in whole blood [6]. The reason that SAM is lower in females is that the upregulation of PEMT by estrogen increases the PEMT flux and that draws down the SAM concentration (simulations not shown).

### Why does folate lower Hcy?

Hyperhomocysteinemia, elevated plasma homocysteine, is a major risk factor for cardiovascular disease, including stroke, myocardial infarction, venous thromboembolism [51], and is also observed in autism [52], renal failure [53], neural tube defects [54], pregnancy complications [55], and some neuropsychiatric diseases [56]. Homocysteine is the product of all transmethylation reactions that use SAM as a methyl donor (five are indicated in Fig. 1). Homocysteine can be remethylated to methionine by MS or BHMT and is converted to cystathionine in the transsulfuration pathway by the enzyme CBS. See Fig. 1.

It has been understood for a long time that increasing folate intake lowers plasma homocysteine [57]. In fact, physicians routinely prescribe folate to patients that show hyperhomocysteineemia [57] and folate fortification of grain and cereal products in the USA lowered plasma homocysteine in the population [58]. The question is why? It would be natural to assume that folate lowers homocysteine because increased total folate increases the concentration of 5mTHF and that speeds up the MS reaction thus lowering Hcy; see Fig. 1. But this explanation is **false** as we will demonstrate. We will then give the correct explanation.

**Fig. 5:**
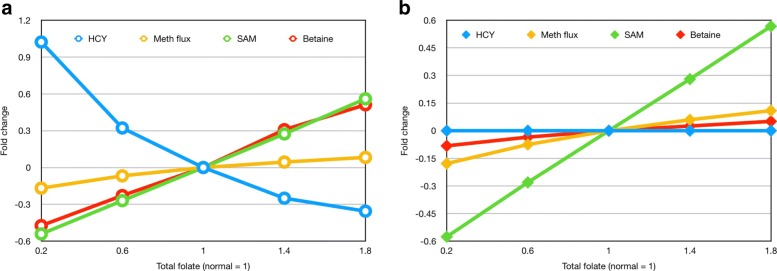
Why does folate lower Hcy? In both panels, total folate is indicated on the x-axis with normal folate = 1. Panel **a** shows simulation results for the concentrations of Hcy (blue), remethylation flux from Hcy to Met (yellow), SAM (green), and betaine (red) in the normal male model as a function of total folate. Hcy decreases substantially as total folate increases. Panel **b** shows simulation results for the same quantities in the normal male if the allosteric effects (red arrows in Fig. 1) of SAM and betaine on CBS are removed. In this case, Hcy concentration is a constant independent of total folate. In both cases the flux from Hcy to Met increases as total folate increases. Thus, Hcy goes down as folate goes up because of the allosteric inhibition of CBS by SAM and Bet

Panel A of Fig. 5 shows the concentrations of Hcy, SAM, and betaine in the male one-carbon model as the level of total folate is varied; folate = 1 is normal. The green curve shows that SAM increases linearly with total folate (as discussed above) and that betaine (the red curve) rises with total folate. As expected, Hcy (blue curve) decreases as total folate increases. The yellow curve is the remethylation flux (from Hcy to Met) which increases slowly as total folate increases. It is this rise in remethylation flux that is credited in the standard argument with lowering Hcy as folate is increased. Panel B shows that this explanation is wrong. In Panel B, we graph the same quantities but we have removed the activation CBS by SAM and the activation of CBS by betaine (indicated by the red arrows in Fig. 1). As one can see in Panel B, the Hcy concentration does not change despite large changes in folate and despite the same changes as in Panel A of remethylation flux. Thus it is not the increased remethylation flux that lowers Hcy, but rather the activations of CBS by SAM and betaine. The intuitive reason that increasing the remethylation flux does not lower Hcy is that all the methyl groups that are carried by the remethylation reactions from Hcy to Met return from SAH to Hcy, and thus do not change the concentration of Hcy.

### Why do women have lower Hcy?

We explained in the previous section that folate supplementation decreases Hcy because it raises the SAM and betaine concentrations. So it is not surprising that betaine supplementation has also been shown to be effective for lowering elevated homocysteine levels [59, 60, 69]. Our discussion of sex differences in choline and betaine in the “Choline and Betaine” section makes it clear why females have lower Hcy than males. Females have approximately twice as much betaine in their liver cells than males and the increased betaine drives the CBS reaction and lowers Hcy. Analogous to the explanation in the previous section, the lowering of Hcy is **not** because betaine increases the flux of the BHMT reaction. It does increase the flux but that is not the reason that Hcy goes down.

Table 5 shows the values of the betaine, Hcy, and SAM concentrations and the velocity of the BHMT reaction (vBHMT) for the male in the mathematical model (column 1). The second column shows the values of the same four quantities for the female. Females have lower Hcy. The third column shows the same four quantities for a simulation for the female where we have made only one change; we removed the activation of CBS by betaine. As one can see, Hcy concentration (1.93) is now even higher than in the male. And, note that this is true even though the velocity of the BHMT reaction has gotten even higher. Thus it is the activation of CBS by betaine that causes Hcy to be lower in females, not the increase in the velocity of the BHMT reaction.

**Table 5:** Betaine, Hcy and SAM simulation concentrations and BHMT simulation velocity (vBHMT) for the male model and the female model

	Male	Female	Female - CBS
Betaine	315	632	562
vBHMT	35.48	40.96	41.19
Hcy	1.80	1.66	1.93
SAM	29.03	25.74	26.12

Female-CBS, the activation of CBS by betaine is removed

We remark that in human epidemiological studies, Hcy is measured in plasma, not in the liver. The relationship between the Hcy concentrations in the liver, other tissues, and the plasma is a complicated issue that we explain in the Discussion.

### Vitamin deficiencies and polymorphisms.

Our studies of OCM using mathematical models have shown that the allosteric interactions (some shown by red arrows in Fig. 1) cause certain substrates and velocities to be quite homeostatic in the face of polymorphisms, vitamin deficiencies, and variation of inputs [15, 16]. For example, the allosteric binding of folate species to folate enzymes makes the folate cycle velocities homeostatic as total folate varies [11]. The long range interactions make the DNA methylation velocity and the Hcy concentration quite stable in the face of variation in methionine input [49, 61]. The TS and AICART velocities, crucial for cell replication, are not affected much by polymorphisms in MS and MTHFR [14]. This is not to say that vitamin deficiencies, changes in input, and polymorphisms have no effects. They do, but the effects may be smaller than one would expect because the entire system compensates. We illustrate these ideas here by discussing the dependence of the choline and homocysteine concentrations on folate, MTHFR polymorphisms, and methionine input.

**Fig. 6:**
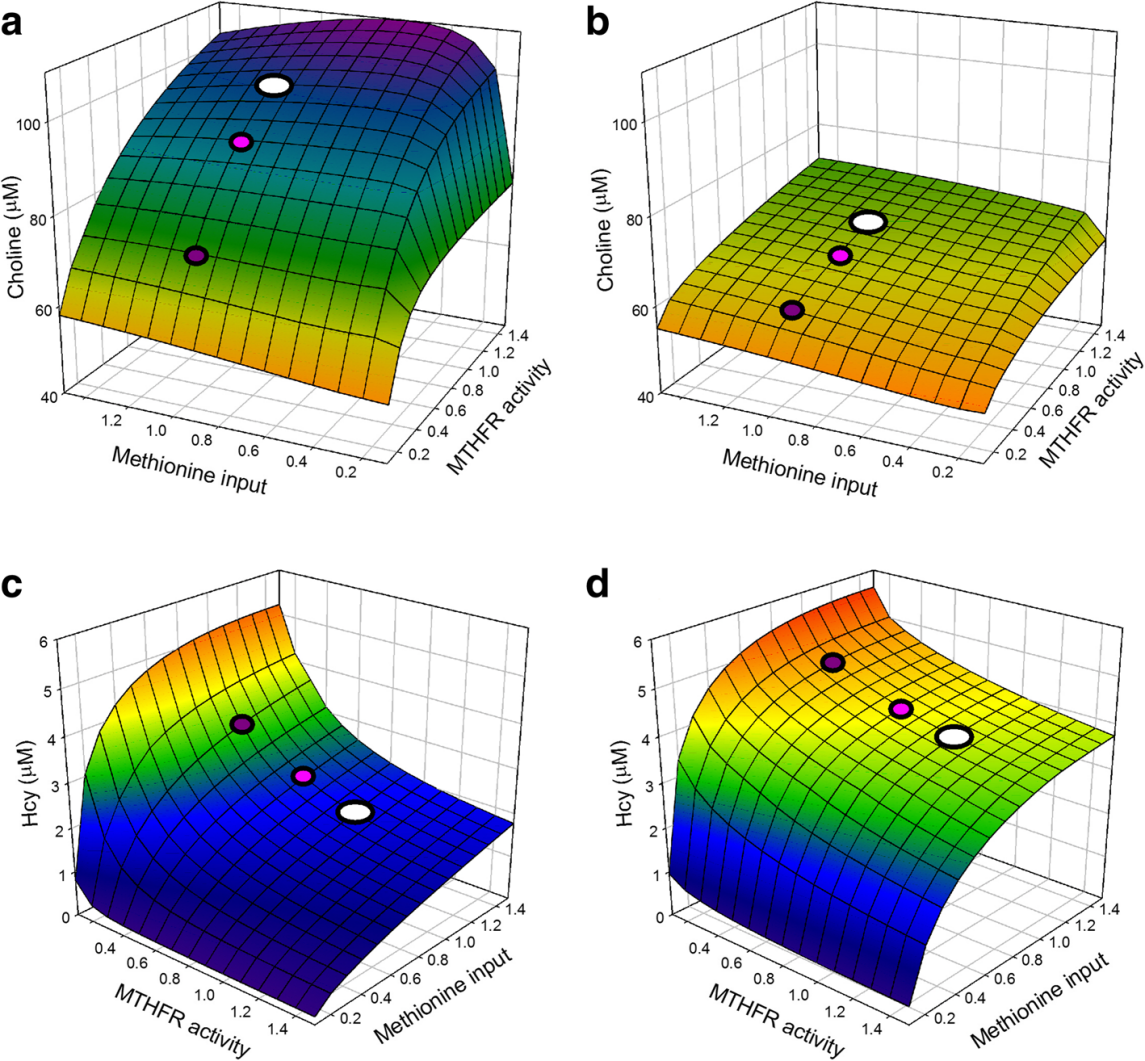
MTHFR polymorphisms, methionine variation, and folate deficiency. Panel **a** is a graph of the how the steady state concentration of choline in the female model depends on MTHFR activity and methionine input. The wild type is shown by the white dot and the two magenta dots correspond to the C677T and A1289C polymorphisms of MTHFR. Panel **b** shows the corresponding surface in case of a folate deficiency that is 20% of normal. Panel **c** shows the homocysteine concentration as a function of MTHFR activity and methionine input. Panel **d** shows the homocysteine surface in the presence of a folate deficiency that is 20% of normal

One might expect that polymorphisms that lower the expression or activity MTHFR would affect choline concentration because when 5mTHF goes down the flux in the MS reaction will go down and the inhibition of GNMT is withdrawn; both of these effects should lower SAM and therefore decrease the flux in the PEMT reaction leading to choline. Panel a of Fig. 6 shows that this is, in fact, the case. The surface shows the choline concentration for the female model as a function of the activity of MTHFR and the methionine input, with 1 indicating “normal” in both cases. The large white dot is the normal steady state. The two magenta dots show the steady states in case of the C677T and A1289C polymorphisms of MTHFR that reduce the activity of MTHFR by 70% and 32%, respectively [37, 38]. Both polymorphisms lower the choline concentration substantially.

Panel b of Fig. 6 shows how the surface changes in the presence of a folate deficiency that is 20% of normal folate. Now wild type, and both polymorphisms give choline concentrations that are below the choline concentration for the C677T polymorphism in Panel a. It is well known that low folate status in the mother is associated with occurrence of neural tube defects [62]. A natural mechanism for this association is that the fetal cells are rapidly dividing and the folate cycle is necessary for duplicating DNA and cell division. What we have shown here gives another connection between low folate status and neural tube defects. Low folate status causes low choline concentrations and choline is required for making fetal cell membranes. The connection between choline status and fetal development was investigated in [63].

In contrast, Panels a and b show that the choline concentration is not very sensitive to methionine input. This is surprising since shouldn’t higher methionine input lead to higher SAM thus increasing the flux on the PEMT-choline pathway? The reason for this homeostasis is as follows. If SAM starts to go up (for example because methionine input increases), then SAM will inhibit BHMT more preventing remethylation of Hcy to Met and SAM will activate CBS more sending more flux down the transsulfuration pathway and both effects limit how much SAM will rise. If SAM starts to fall (for example, because MTHFR loses activity) then the inhibition of BHMT is withdrawn and the activation of CBS is withdrawn thus remethylating more Hcy to Met and limiting the decline in SAM. As a result, over wide ranges, methionine input does not affect choline concentration very much. Since methionine input changes a lot during the day because of meals this may be an important evolutionary adaptation.

Panel c shows the homocysteine concentration at steady state in the female model as a function of MTHFR activity and methionine input. As above, the white dot is wild type and the magenta dots indicate the steady state concentration of Hcy in the presence of the polymorphisms. Both polymorphisms substantially raise Hcy concentration. However, as can be seen in Panel D, in the presence of a folate deficiency that is 20% of normal, the Hcy concentration of even the wild type is higher than those caused by the polymorphisms in case of no folate deficiency (Panel c). This is another indication of the importance of folate. The mechanism by which folate affects Hcy concentration is discussed in the “Why does folate lower Hcy?” section, above. Notice that the Hcy concentration is not very dependent on methionine input near the normal input though it does plunge to zero as methionine input goes to zero. This is further discussed in [61].

## Discussion

The purpose of this study was to understand sex differences in one-carbon metabolism and for that purpose we added the synthesis pathway of choline and betaine to a previous mathematical model [17]. Although we base our models on the underlying physiology and biochemistry, no model can represent the full complexity of cellular systems that involve metabolism, gene expression of enzymes, and interactions between them. For example, we modeled BAH, betaine aldehyde dehydrogenase, with simple Michaelis-Menten kinetics when, in fact, BAH shows substrate inhibition by betaine aldehyde, product inhibition by betaine, and inhibition by choline [64]. CBS shows substrate inhibition by Hcy and is inhibited by cystathionine; neither effect is included in our model. We have not included the folate cycle in the mitochondria [18], nor the full transsulfuration pathway even though glutathione disulfide affects MAT-I in the methionine cycle [19]. Nevertheless, we have been able to use our model to understand how the enzyme expression differences between males and females cause the concentration differences observed. As we discussed in “Sex differences” section, gene expression differences between males and females are known to be caused by the sex steroids and by the pattern of growth hormone secretion [4, 41–44, 68, 69].

Recently, a large scale semi-quantitative model of sex differences in liver metabolism has been published [45–47] based on an earlier model of male liver metabolism [65]. The authors show that this model, which includes both sex hormone and growth hormone effects, allows a detailed insight into sex-dependent liver pathologies and they use the model to identify the most important sex-dependent pathways involved in non-alcoholic fatty liver disease. Our work here complements this work by focusing on sex differences in folate and methionine metabolism and methylation reactions.

The enzyme PEMT catalyzes the production of PC, the first substrate on the pathway that synthesizes choline and betaine. Choline and betaine are essential nutrients obtained from the diet or from the synthesis pathway on the right side of Fig. 1. Choline is needed for neurotransmitter synthesis, the construction of cell membranes, cell-membrane signaling, lipid transport, and methyl-group metabolism. It plays important roles in brain and memory development in the fetus and appears to decrease the risk of the development of neural tube defects [63]. Betaine, derived from dietary choline, is an osmolyte protecting cells, proteins, and enzymes from environmental stress. Betaine is also a methyl donor, participating in remethylation in the methionine cycle.

The upregulation of PEMT by estrogen in women of child-bearing years greatly increases the concentrations of choline and betaine as we saw in the “Choline and Betaine” section. But why? Maternal choline is important for neural tube closure in the fetus and for neurodevelopment in the fetal hippocampus, which effects memory. In fact, pregnant subjects fed 4 times the dietary levels of choline [63] had offspring with a 30% enhanced visuospatial and auditory memory, and these enhanced functions did not decrease as they aged. Thus, it seems extremely likely that the upregulation of PEMT by estrogen is an evolutionary mechanism for ensuring large choline supplies for the fetus and the mother. The evolutionary reasons for the other expression level differences caused by the sex hormones remain uncertain.

It is important to remember that the mathematical model in this paper is for liver one-carbon metabolism. We explained, in the “Why does folate lower Hcy?” section and the “Why do women have lower Hcy?” section, why folate lowers Hcy and why females have lower Hcy than males in the liver. However, Hcy levels in humans are measured in the blood or plasma. Although it is commonly thought that blood and plasma levels reflect liver values, we have shown with a whole body model of folate metabolism that that is not true [20]. Blood and plasma levels are driven by tissue levels, not liver levels of Hcy. Tissues like muscle do not express BHMT and show low expression of CBS, so they have high levels of Hcy, which is exported to the plasma where much is taken up by the liver and catabolized in the liver transsulfuration pathway [20]. Women have lower blood and plasma levels of Hcy because they have higher betaine levels in tissues (because estrogen upregulates PEMT) and betaine activates CBS. Since CBS is the main removal mechanism for Hcy in tissues, one would expect the activation of CBS to be even more important than in the liver. The point is that the relatively small liver decrease of Hcy in females that we have found (1.80 to 1.66) does not reflect the larger decreases found in the clinic (Table 2) because those decreases are driven by the tissues. So the explanation is still the same but size of the decreases in the blood and plasma may be different.

This study uses mathematical modeling to investigate sex differences in one carbon metabolism, but it is only a first step because there are many other issues to be analyzed. Since estrogen levels vary during the menstrual cycle, our results suggest that Hcy levels should vary too, and they do [66, 67]. Kalhan and co-workers have shown the transmethylation, remethylation, and transsulfuration fluxes vary greatly between the three trimesters of pregnancy [2, 3]. And, the enzymes of one-carbon metabolism are also affected by insulin and glucose levels [10] that can change dramatically during pregnancy. These phenomena will be the subject of future work.

## Conclusions.

We have created a new mathematical model of one-carbon metabolism in the liver including the synthesis pathway for choline and betaine. The model was used to understand how observed enzyme expression differences lead to metabolite concentration differences. In particular, we explained why women have lower S-andenosylmethionine, lower homocysteine, and higher choline and betaine. We give a new explanation of the well known phenomenon that folate supplementation lowers homocysteine and show how the model can be used to investigate the effects of vitamin deficiencies, gene polymorphisms, and nutrient input changes. Future work will explore how one-carbon metabolism changes during pregnancy.

## Additional file

Additional File 1 Supplementary Material for Sex Differences in One-Carbon Metabolism. (PDF 710 kb)
